# Pain in Veterans with COPD: relationship with physical activity and exercise capacity

**DOI:** 10.1186/s12890-021-01601-8

**Published:** 2021-07-15

**Authors:** Rebecca A. Raphaely, Maria A. Mongiardo, Rebekah L. Goldstein, Stephanie A. Robinson, Emily S. Wan, Marilyn L. Moy

**Affiliations:** 1 Pulmonary and Critical Care Medicine Section, VA Boston Healthcare System, 1400 VFW Parkway, Mail Code 111P, Boston, MA 02132 USA; 2 Department of Pulmonary and Critical Care, Massachusetts General Hospital, Boston, MA USA; 3 Department of Pulmonary and Critical Care, Beth Israel Deaconess Medical Center, Boston, MA USA; 4 Harvard Medical School, Boston, MA USA; 5 VA Bedford Healthcare Systems, Bldg 70, 200 Springs Rd, Bedford, MA 01732 USA; 6 Boston University School of Medicine, Boston, MA USA; 7 Channing Division of Network Medicine, Brigham and Women’s Hospital, Boston, MA USA

**Keywords:** Chronic Obstructive Pulmonary Disease (COPD), Pain, Physical activity (PA), Exercise capacity, 6-Minute walk test (6MWT), Physical activity intervention

## Abstract

**Background:**

Pain is a common but underappreciated symptom experienced by people with Chronic Obstructive Pulmonary Disease (COPD). The relationships between pain and physical activity (PA) and exercise capacity are poorly understood.

**Methods:**

This retrospective secondary analysis includes three cohorts of Veterans with COPD who participated in longitudinal studies evaluating PA and exercise capacity with objective measures of daily step counts and 6-min walk test (6MWT) distance, respectively. Pain was assessed using the bodily pain domain of the Veterans RAND-36. In two cohorts, participants were randomly assigned to a web-based, pedometer-mediated PA intervention which has previously been demonstrated to improve PA.

**Results:**

Three-hundred and seventy-three (373) unique study participants were included in this analysis. Eighty-three percent (n = 311) of the population reported at least mild pain and/or at least a little bit of interference due to pain at baseline. Cross-sectionally, greater bodily pain was associated with lower 6MWT distance (β = 0.51; 95% CI 0.20, 0.82; p = 0.0013). Longitudinally, worsening bodily pain was associated with a decline in 6MWT distance (β = 0.30; 95% CI 0.03, 0.58; p = 0.0312). There was no association between baseline bodily pain and baseline daily step counts, baseline bodily pain and change in PA, or change in bodily pain and change in PA. Compared to usual care, our PA intervention improved bodily pain scores (β = 6.17; 95% CI 1.84, 10.45; p = 0.0054). Bodily pain scores did not affect the impact of the intervention on daily step counts.

**Conclusion:**

Pain is highly prevalent and significantly associated with lower exercise capacity among Veterans with COPD. Worsening pain co-occurred with decline in exercise capacity but not PA. Our intervention reduced pain, although pain did not affect the impact of the intervention on PA.

## Introduction

Despite optimal medical management, people with Chronic Obstructive Pulmonary Disease (COPD) experience a substantial physical and psychological symptom burden [1–3]. Although dyspnea is the most commonly reported symptom, recent studies suggests that pain is also highly prevalent but less well-recognized in this population [2–4]. Among patients with COPD, the pooled prevalence of pain is estimated to be 66% with a range of 45–96%, depending on the sample size, study design, and definition of pain used [4–12]. Compared to a population with chronic medical conditions matched for gender, age, and comorbidities, a significantly higher percentage of people with COPD have a diagnosis of pain [11, 12]. Epidemiological studies also suggest that people with COPD have higher rates of chronic opiate and non-opiate pain medication prescription compared to age and gender match controls, supporting the assertion that pain is more prevalent in this population [12, 13].

Promotion of exercise and physical activity (PA) is the standard of care in persons with COPD [14–16]. In the COPD population, greater PA is associated with improved health related quality of life (HRQL), reduced risk of acute exacerbations and hospitalizations, and greater survival, independent of lung function [14–20]. Three cross-sectional studies, and one study with short-term follow-up, have demonstrated an association between pain and decreased levels of PA (self-report, surveys, standing time) and exercise capacity, as measured by the 6-min walk test (6MWT), among patients with COPD [21–24]. There are no studies to date, however, evaluating these relationships with direct measures of PA in persons with COPD ready to engage in PA. In addition, understanding the prevalence of pain in this population and studying how technology-based PA interventions impact pain are critical to effectively implement the GOLD recommendation to promote PA in all persons with stable COPD [14].

We have previously studied Veterans with COPD who self-selected to enroll in research studies using accelerometers and pedometers to directly measure PA [18, 25–27]. In one observational cohort study, we measured daily step counts over 14 days at two time points, 3 months apart, and evaluated the association between PA and 6MWT, HRQL, and acute exacerbations [18, 25]. In two separate studies, we demonstrated the efficacy of a web-based, pedometer-mediated intervention to increase PA [26, 27]. In this retrospective secondary analysis, we combined these three well-characterized cohorts in order to (1) understand the prevalence of pain in persons with COPD who are ready to engage in PA, (2) evaluate the cross-sectional and longitudinal associations between pain and exercise capacity and PA, (3) examine the impact of our web-based pedometer-mediated PA intervention on pain, and (4) assess the impact of pain on the response to our PA intervention.

## Materials and methods

### Study participants

The study population consisted of three cohorts of Veterans with COPD (n = 375) who volunteered to participate in PA studies. Cohort 1 (n = 163) was an observational study including people recruited from pulmonary clinics at the Veterans Affairs (VA) Boston Healthcare System between 2009 and 2011 [18, 25]. Cohort 2 (n = 108) included Veterans enrolled from pulmonary clinics at VA Boston from 2012–2015 for a randomized controlled trial (RCT) (NCT01772082) comparing a web-based, pedometer-mediated PA intervention to pedometer alone [26]. Cohort 3 (n = 104) included people enrolled from VA Boston from 2015 to 2019 for a RCT in which participants were assigned to the technology-based PA intervention or usual care (NCT02099799) [27]. People randomized to the intervention arm participated in the same web-based, pedometer-mediated intervention (Table 1). All protocols were approved by the VA Boston Healthcare System Institutional Review Board on Human Subjects Research (Protocol #1961, Protocol #2328, Protocol #2791), and written informed consent was obtained from participants. In cases where study participants enrolled in more than one trial, only the most recent trial data were used so that all study participants are unique. Of note, participants in cohorts 1 and 2 used the Omron HJ-720 ITC pedometer while those in cohort 3 used the FitBit Zip pedometer because the Omron HJ-720 ITC was discontinued. The accuracy of both pedometers in people with COPD has been confirmed in previous studies [31, 32].

**Table 1:** Characteristics of three studies of Veterans with COPD and an interest in physical activity

	Cohort 1 [18, 25]	Cohort 2 [26]	Cohort 3 [27]
Study participants	163	108	104
Study design	Observational Cohort	Randomized Controlled Trial	
Study Sites	VA Boston	VA Boston	VA Boston, VA Birmingham*
Recruitment period	2009–2011	2012–2015	2015–2019
Study duration	3 months	3 months	6 months
Intervention group	NA	Web-based pedometer-mediated physical activity program	
Comparison group	NA	Pedometer	Usual care
Pedometer type	Omron HJ-720 ITC	Omron HJ-720 ITC	FitBit Zip
Valid pedometer wear days for step counts	≥ 200 steps and ≥ 8 h of wear time	≥ 100 steps and ≥ 8 h of wear time	≥ 200 steps
Minimum days assess for step counts	≥ 5	≥ 5	≥ 5

*Only VA Boston participants are included in the analysis. VA Birmingham participants were not consented for reuse of data

Analyses of all data are approved by VA Boston IRB #2999 Pulmonary Research Data Repository. Either participants provided written consent, or the IRB approved a waiver of HIPAA authorization for use of repository data. Study was carried out in accordance with the Declaration of Helsinki.

### Inclusion/exclusion criteria

Study participants were ≥ 40 years-old with a diagnosis of COPD based on a smoking history ≥ 10 pack-years and a forced expiratory volume in the first second (FEV_1_) to forced expiratory capacity (FVC) ratio of ≤ 0.70 or emphysema on chest computed tomography. Exclusion criteria included unstable cardiovascular disease, acute exacerbation of COPD < 4 weeks prior to enrollment, and inability to ambulate.

### Intervention

Study participants in cohort 2 and cohort 3 were assigned to the same PA intervention consisting of a pedometer plus a website that provides goal setting, feedback, motivational messages, educational content, and social support. This intervention has previously been shown to increase PA and improve HRQL at 3 and 6 months [26, 27]. To evaluate the impact of the PA intervention on pain, study participants were divided into groups based on participation in the PA intervention. Group 1, those assigned to the intervention (n = 111), consisted of participants in cohort 2 (n = 57) and cohort 3 (n = 54). Group 2, those who did not use the intervention (n = 264), consisted of participants in cohort 1 (n = 163), cohort 2 (n = 47), and cohort 3 (n = 54). Among the participants in group 2, those from cohort 1 and cohort 3 (n = 217) received usual care while those from cohort 2 (n = 47) used a pedometer throughout the study period.

### Outcomes

Participants completed spirometry, 6MWT, assessment of PA as daily step counts, Veterans RAND-36 Item Health Survey (VR-36), modified Medical Research Council dyspnea scale (mMRC), and self-reported demographics and medical history at the time of enrollment and at 3-months follow up.

### Pain

Pain was assessed using the bodily pain domain of the VR-36 questionnaire [28, 29]. The bodily pain domain includes two questions assessing (1) pain intensity and (2) pain interference, over the preceding 4 weeks. Responses were evaluated using a standardized composite scoring system with scores ranging from 0 to 100 [29]. A score of ‘100’ represents no pain while a score of ‘0’ represents very severe pain causing extreme interference with activities. The minimal clinically important difference (MCID) for the bodily pain domain was extrapolated from a COPD population evaluated with Short Form-36 which uses the same two questions to assess pain severity and interference [30]. The MCID for a small improvement in pain corresponds to a 10-point increase in the bodily pain score, a moderate improvement corresponds to a 20-point increase, and a large improvement correspond to a 30-point increase [30].

### Physical activity

Physical activity was measured by average daily step counts assessed objectively using pedometers: Omron HJ-720 ITC pedometer (cohort 1 and 2) and Fitbit Zip (cohort 3). We have previously demonstrated that pedometers can accurately capture daily step counts among Veterans with COPD [31, 32].

Criteria for valid wear days are detailed in Table 1. All participants in both groups were blinded to step-count feedback during a 7-day baseline collection period prior to randomization using an opaque sticker covering the pedometer face (preventing feedback of daily step counts). After the baseline collection period, study participants were not blinded to step-count data. Group 1 participants had access to pedometers throughout the study. Monitoring of group 2 participants varied by study. Participants from cohort 2 had access to an Omron pedometer throughout the study and were not blinded to step counts after initial baseline data collection. Participants from cohort 3 had access to the study pedometer only during the baseline collection period and for 14-days after the 3-month study visit. The MCID for daily step counts in persons with COPD ranges from 350 to 1100 steps/day [33, 34].

### Exercise capacity

Exercise capacity was assessed with maximal distance walked on the 6MWT in accordance with the American Thoracic Society/European Respiratory Society (ATS/ERS) guidelines, without a practice walk [35]. In this assessment, patients determined the exercise intensity and speed, were allowed to rest, and used supplemental oxygen, if prescribed for activity. The MCID for 6MWT distance is 30–54 m [36, 37].

### Pulmonary function

Spirometry was performed at enrollment according to ATS guidelines [38]. FEV_1_ percent predicted was calculated according to Hankinson’s references [39].

### Dyspnea

Dyspnea was assessed at baseline and 3 months with the mMRC dyspnea scale. Responses range from 0 to 4 with higher scores representing more severe dyspnea [40]. The MCID has been reported to be one unit [41]. Dyspnea was dichotomized (≤ 2 or > 3) for analysis.

### Statistical analysis

Of the 375 study participants in the combined cohorts, two people were excluded because of incomplete pain assessment at enrollment. Summary statistics are presented as means ± standard deviations for continuous variables and frequencies for categorical or ordinal variables. Generalized linear regression models (PROC GLM) were implemented to explore cross-sectional and longitudinal relationships between baseline bodily pain scores, 6MWT distance, and daily step counts. Generalized linear regression models were also used to test the association between the change in bodily pain scores, change in 6MWT distance, and change in daily step counts. Change in bodily pain was evaluated as both a continuous and dichotomous variable based on the MCID. Multivariable models were adjusted for age, gender, percent predicted FEV_1_, body mass index (BMI), dyspnea (mMRC score), and cohort. These covariates were selected based on known associations with the outcomes and in order to adjust for possible differences between cohorts. Covariates were evaluated for collinearity prior to inclusion. Models evaluating change in 6MWT distance and change in daily step counts were also adjusted for group and baseline 6MWT distance and daily step counts when indicated. Season of enrollment was included as a covariate for models with daily step counts as the outcome given established association between season and step counts.

Differences between groups were evaluated with unpaired t-tests or chi-square tests. The impact of our PA intervention on bodily pain score was evaluated using a generalized linear regression model adjusting for age, percent predicted FEV_1_, BMI, dyspnea, and cohort. Finally, given our previous results demonstrating that our PA intervention is associated with a significant increase in daily step counts [26, 27], we evaluated pain as an effect modifier for the impact of the PA intervention on daily step counts using an interaction term (group*bodily pain score). All analyses were performed in SAS software version 9.4 (Cary, NC, USA).

## Results

### Prevalence of pain

**Fig. 1:**
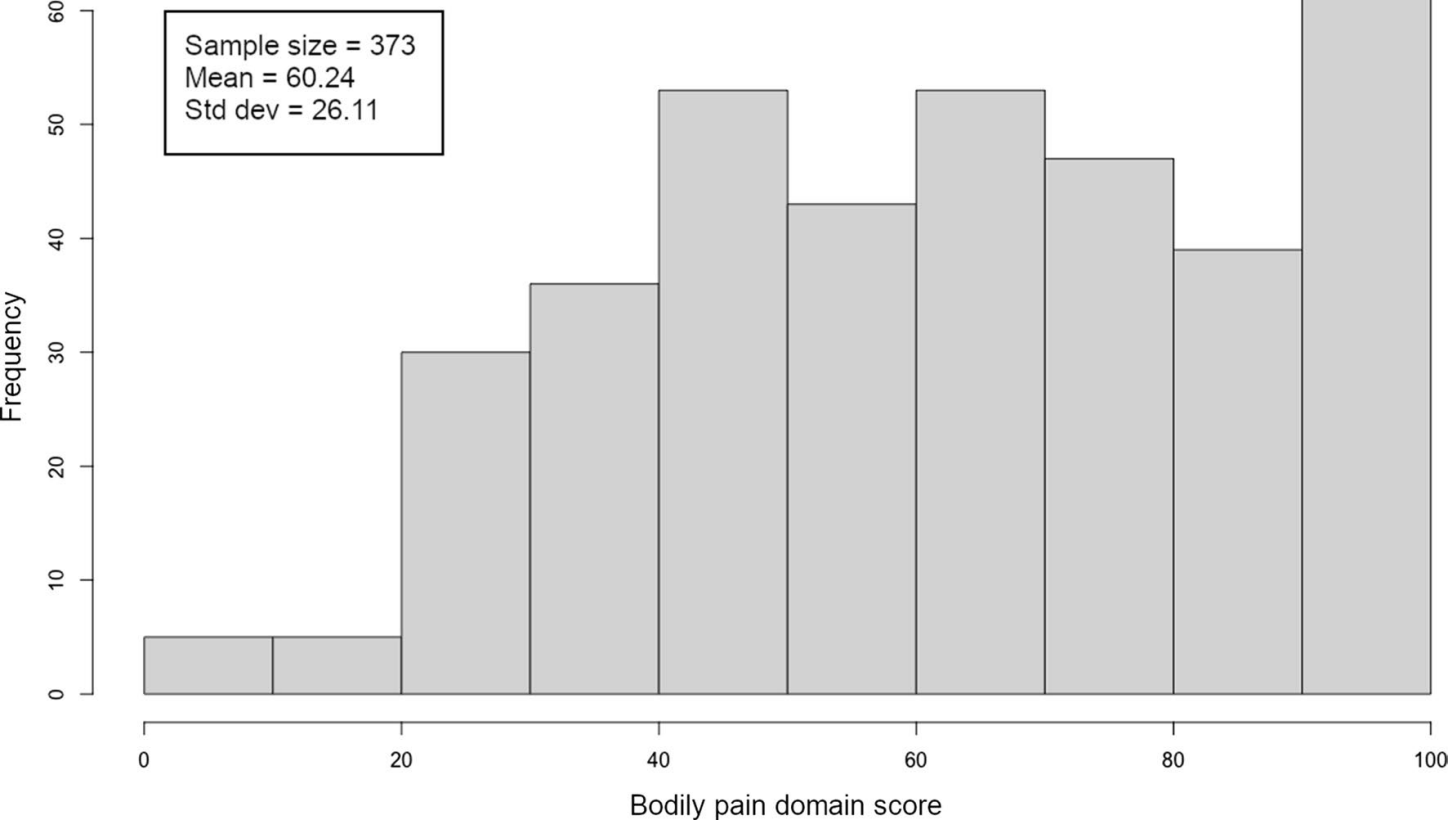
Distribution of bodily pain scores from VR-36 survey. Histogram demonstrating the distribution of bodily pain scores evaluated with the VR-36 survey and assessing symptoms in the four weeks prior to study enrollment. A pain score of ‘100’ (n = 62) corresponds to no pain while a score of ‘0’ (n = 4) indicates very severe pain causing extreme interference with daily activities

For the combined cohort of Veterans interested in engaging in PA, 98% of participants were male with an average age of 70 ± 8 years, percent predicted FEV_1_ of 59 ± 21, and BMI of  29 ± 6 kg/m^2^. Baseline average 6MWT distance was 376 ± 96 m and daily step counts were 3,167 ± 2,364 steps/day. There was no significant difference between age, percent predicted FEV_1_, and BMI between the three cohorts included in the study population (Table 2). The range and distribution of pain scores are shown in Fig. 1 with a score of 100 denoting no pain and a score of 0 indicating very severe pain causing extreme interference with activity. Eighty-three percent of the participants (311/373) reported at least mild pain and/or at least a little bit of interference due to pain at enrollment, with the average bodily pain score of 60 ± 26 points.

**Table 2:** Characteristics of Veterans with COPD who volunteered for PA studies

	Group 1 Exposed to web-based PA intervention	Group 2 Unexposed to web-based PA intervention	Combined cohorts	*P*-value
N	111	262	373	
Age—year (SD)	69 (8)	71 (8)	70 (8)	0.0545
Male gender—no. (%)	108 (97)	256 (98)	364 (98)	0.8123
White race—no. (%)	103 (93)	243 (93)	346 (93)	0.8984
Married no. (%)	52 (47)	129 (49)	181 (49)	0.5047
BMI—kg/m^2^ (SD)	30 (6)	29 (6)	29 (6)	0.0871
Pack-years (SD) (n = 369)	58 (35)	64 (39)	62 (38)	0.1403
FEV_1_% predicted—avg (SD) (n = 367)	62 (21)	58 (21)	59 (21)	0.0833
Supplemental oxygen use—no. (%)	22 (20)	60 (23)	82 (22)	0.5341
MMRC—no. (%)				0.1957
0–2	74 (67)	156 (60)	230 (62)	
3–4	37 (33)	106 (40)	143 (38)	
6MWT distance—meters (SD)				
Baseline	380.54 (89.61)	373.75 (98.57)	375.77 (95.93)	0.5327
3 months (n = 283)	385.50 (95.65)	377.70 (102.60)	380.48 (100.06)	0.5298
∆ 6MWT (n = 283)	0.47 (47.61)	0.17 (44.33)	0.28 (45.44)	0.9576
Daily step counts—steps (SD)				
Baseline	3296 (2361)	3112 (2367)	3167 (2364)	0.4927
3 months (n = 275)	4024 (2479)	3054 (2302)	3403 (2408)	0.0013*
∆ steps (n = 275)	678 (1857)	− 180 (1470)	129 (1669)	0.0001*
Bodily pain—avg (SD)				
Baseline	61.11 (25.14)	59.87 (26.54)	60.24 (26.11)	0.6770
3 months (n = 284)	65.73 (22.99)	60.47 (24.63)	62.36 (24.15)	0.0783
∆ Bodily pain (n = 284)	3.12 (22.37)	− 2.64 (18.07)	− 0.57 (19.88)	0.0273*

Study participants were divided into two groups based on exposure to a web-based pedometer-mediated PA intervention. Group 1 consists of study participants exposed to a PA intervention while group 2 consists of study participants who were not randomized to undergo a PA intervention

*Indicates a significant p-value less than or equal to 0.05

### Cross-sectional relationships between pain, 6MWT distance, and daily step counts

**Fig. 2:**
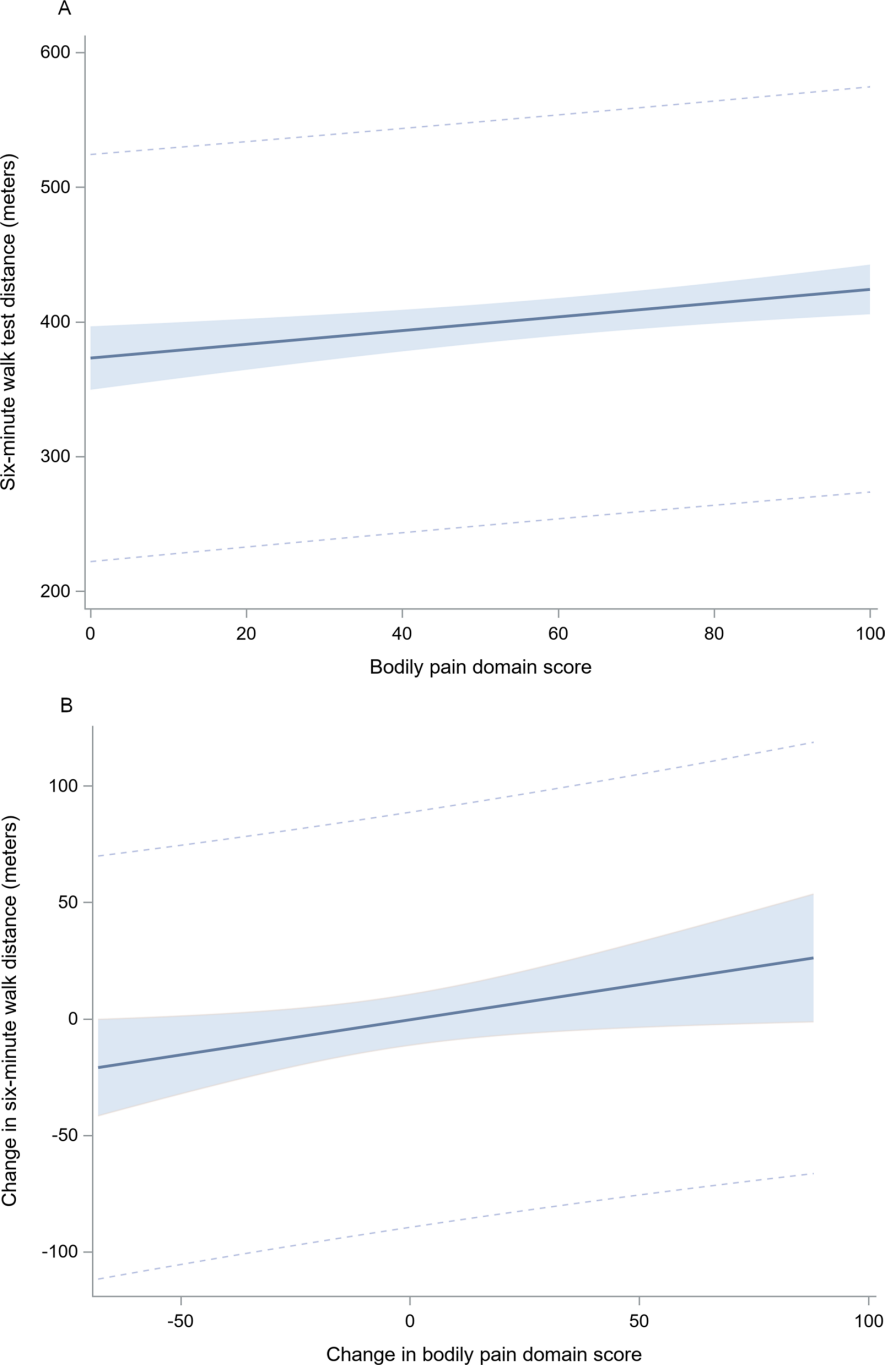
Relationships between pain and exercise capacity cross-sectionally (**A**) and longitudinally (**B**). Generalized multivariate linear regression models with 95% confidence interval adjusted for BMI, percent predicted FEV_1_, and cohort. A bodily pain score of ‘100’ represents no pain while a score of ‘0’ represents severe pain causing extreme interference. A change in pain score > 0 indicates improved pain. **A** Represents results of a model evaluating the cross-sectional relationship between bodily pain score and 6MWT distance both measured at baseline. **B** Represents results of a model evaluating the longitudinal relationship between the change in bodily pain and 6MWT

At baseline, higher bodily pain scores (indicative of less pain severity and interference) were positively associated with higher 6MWT distance. In this model, a 10-point increase in bodily pain score (less pain) was associated with a 5.1-m increase in 6MWT distance (p = 0.0013) (Table 3, Fig. 2A). Accordingly, a 58-point difference in bodily pain score would achieve the MCID in 6MWT distance (≥ 30 m) [36, 37]. There was no significant relationship between baseline bodily pain score and daily step counts.

**Table 3:** Relationship between pain and physical activity and exercise capacity

Outcome	Predictor	Beta coefficient (95% Confidence Interval)	*P*-value
Baseline steps	Baseline pain	5.85 (− 2.67, 14.37)	0.1178
Change in steps	Baseline pain	3.05 (− 4.16, 10.25)	0.4061
Change in steps	Change in pain	3.11 (− 6.10, 12.31)	0.5065
Baseline 6MWT	Baseline pain	0.51 (0.20, 0.82)	0.0013
Change in 6MWT	Baseline pain	− 0.08 (− 0.30, 0.14)	0.4617
Change in 6MWT	Change in pain	0.30 (0.03, 0.58)	0.0312

Generalized linear regression models evaluating relationships between bodily pain score, change in bodily pain score, daily step counts, change in daily step counts, 6MWT distance, and change in 6MWT distance. Models adjusted for age, BMI, percent predicted FEV_1_, dyspnea, and cohort. When relevant, additional covariates for group, season at time of enrollment, and baseline 6MWT distance or baseline daily step counts were included. Steps indicate average daily step counts and 6MWT indicates 6MWT distance as measured in meters. Unit of change for bodily pain is 1 point

### Longitudinal relationships between pain, 6MWT distance, and daily step counts

At 3 months, 87 (23%) study participants were lost to follow-up. Compared to people who participated in a study for 3 months those lost to follow-up had worse pain (51 ± 25 vs. 63 ± 26) (p = 0.0003) and were more likely to be from group 2 (n = 79, 30%) than group 1 (n = 8, 7%, p < 0.0001). Age, BMI, mMRC dyspnea scale, and percent predicted FEV_1_ did not significantly differ between those who completed the study and those lost to follow-up.

Among the participants with complete data, baseline bodily pain score did not predict change in either 6WMT distance or daily step counts, adjusting for exposure to the PA intervention. However, an improvement in bodily pain score was positively and significantly associated with an increased 6MWT distance over the 3-months period in the total population when adjusting for group. A 10-point improvement in bodily pain score was associated with a 3.0-m increase in 6WMT distance over 3 months (p = 0.0312) (Table 3, Fig. 2B).

### Impact of web-based PA intervention on pain

Demographics and baseline bodily pain score, daily step counts, and 6MWT distance did not significantly differ between the two groups (Table 2). Among the 283 participants who had a complete pain assessment at 3 months, use of the PA intervention was associated with a significant decrease in the bodily pain score, compared to the group who received usual care or pedometer. Participants in group 1, on average, had a 6.17-point increase in bodily pain score (corresponding to reduced pain severity and/or interference) compared to those in group 2 (Table 2). Evaluating bodily pain score as a dichotomous variable for a small improvement (change ≥ 10 points vs. < 10 points) 32% (n = 35) of group 1 participants experienced at least a small improvement of pain while only 15% (n = 38) of group 2 participants experienced the same benefit. Accordingly, participation in the PA intervention was associated with 2.71 times the odds of having at least a small improvement in bodily pain score over 3 months (OR 2.71, 95% CI 1.60, 4.60; p = 0.0002) [30].

### Baseline pain and response to PA intervention

As previously published in two separate RCTs, participants assigned to our PA intervention experienced a significantly greater improvement in daily step counts compared to those who did not use the intervention [18, 25–27]. In this retrospective analysis of combined datasets, participants in group 1 improved their daily step counts compared to those in group 2 (β = 705 steps/day, 95% CI 261, 1.148; p = 0.0020). Baseline bodily pain score, however, was not a significant predictor for change in daily step counts (β = 3.05 ± 3.66; 95% CI − 4.16, 10.25, p = 0.4061) (Table 3). Furthermore, baseline bodily pain score did not significantly modify the impact of the PA intervention on daily step counts as examined using an interaction term (β = 5.91; 95% CI − 6.15, 17.98; p = 0.3353).

## Discussion

This secondary analysis of persons with COPD who volunteered to participate in PA studies shows that pain is not only highly prevalent in this population, but it is also associated with reduced exercise capacity. Cross-sectionally, lower composite pain scores (worse pain), incorporating pain severity and interference, are associated with lower 6MWT distances, and worsening pain over time is associated with a decline in 6MWT distance. Baseline pain was not, however, significantly associated with baseline step counts, and pain did not modify the impact of a technology-based PA program to increase daily step counts. Importantly, our technology-based PA program reduced pain in persons with COPD.

Our results support previous studies which suggest that pain is highly prevalent among people with COPD and build on findings that pain may be negatively associated with activity and exercise [21–24]. The prevalence of pain (83%) in our population, was at the upper limit of that reported in the literature, 45–85% [4]. Furthermore, the average bodily pain score in our population (60 ± 26) was lower than the average score in a non-VA population with chronic medical and psychiatric conditions (71 ± 25) suggesting greater pain intensity and interference in our COPD study population [42]. In fact, 35% of participants had a bodily pain score lower than one SD from the general population mean (< 45 points), highlighting that pain is common and severe even among those interested in PA. Like previous studies our results demonstrates an association between pain and exercise capacity, however, in contrast to the literature, our work does not support an association between pain and PA. This is likely related to our larger sample size and the use of directly rather than indirectly measured PA.

Our work advances the literature by assessing the longitudinal associations between pain and directly observed PA in patients with COPD and evaluating the relationship between pain and a technology-based PA intervention. To date, one prospective case–control study, by Lee et al., has evaluated the impact of an exercise program on pain [24]. In that study, among people with COPD and chronic pain, a traditional PR program was not associated with change in pain. In contrast, our study demonstrates that a technology-based PA program can reduce pain, underscoring that pain is not necessarily a reason to refrain from PA and exercise counseling in persons with COPD [43, 44]. The study by Lee et al. further demonstrated that the response to PR, measured by 6MWT distance, did not vary based on the presence of pain [24]. However, our results indicate that pain does not modify the impact of a technology-based intervention to increase PA (the primary outcome for that intervention), although pain was significantly associated with 6MWT distance. These results suggest that people with pain benefit from exercise interventions. These findings are important in the COVID-19 era since technology interventions are needed to support COPD patients who must remain physically active while socially distancing. They also support that pain should be routinely evaluated and treated as part of exercise counseling for patients with COPD [43].

Interestingly, despite a significant association between exercise capacity and pain, we did not observe an association between pain and directly measured PA in our relatively large study population. There are several possible explanations. In this retrospective study, we lacked data regarding use of analgesic pain medications and other therapeutic interventions, including massage and heating/cooling pads. It is possible that patients with pain were treating symptoms at home thus mitigating the impact of pain on daily step counts but not 6MWT distance which was assessed episodically in the clinic. Differences in measurement techniques for exercise capacity and PA may also explain the differential impact of pain. Exercise capacity is evaluated via the 6MWT performed under standardized conditions, while daily step counts are measured with a pedometer worn throughout the day in the patient’s home where environmental and psychosocial factors may mitigate the impact of pain [45–48].

Strengths of our study include the large sample size and use of well-described cohorts with rigorously assessed, repeated, and objective measures of PA and exercise capacity. Limitations are inherent to the study design of a retrospective secondary analysis. Based on the available data, we assessed pain using the VR-36 survey. Although the domain evaluates both pain severity and interference, a more comprehensive and detailed survey that has previously been validated in people with COPD, like the Brief Pain Inventory, would likely be helpful in assessing pain with more granularity [49]. Our population consisted of older, Caucasian men recruited from a single geographical location, limiting generalizability and underscoring the need for research including a more diverse population especially given that the perception of pain may be mediated through gender and cultural perspectives. Finally, our study design, in which we recruited participants interested in participating in PA programs, selected for a population with relatively tolerable pain and significant motivation to exercise. This is also supported by the differential loss to follow up based on the observation that participants with greater pain were less likely to complete the study. This may suggest that our results cannot necessarily be extrapolated to people with more severe or debilitating pain who are likely minimally active. Despite this, the relatively low rate of attrition in the intervention group compared to the usual care/pedometer group (7% vs. 30%) combined with significant improvement in pain observed in the intervention group suggest that participation in a PA program is overall beneficial to those with pain.

## Conclusions

Our study demonstrates a high prevalence of pain and a significant association between pain and exercise capacity among patients with COPD interested in engaging in PA. A technology-based PA intervention may be a nonpharmacological treatment of pain. Identifying and treating pain should be routine parts of exercise counseling in COPD.

## Data Availability

The datasets generated and/or analyzed during the current study are not publicly available according to rules of the Veterans Health Administration but are available from the corresponding author upon reasonable written request and approval by the designated Privacy Officer and Information Security Officer.

## Abbreviations

ATS: American Thoracic Society
BMI: Body mass index
CI: Confidence interval
COPD: Chronic Obstructive Pulmonary Disease
ERS: European Respiratory Society
FEV_1_: Forced expiratory volume in the first second
FVC: Forced expiratory capacity
GOLD: Global Initiative for Obstructive Lung Disease
HRQL: Health related quality of life
MCID: Minimally clinically important difference
mMRC: Modified Medical Research Council
PA: Physical activity
PR: Pulmonary rehabilitation
RCT: Randomized controlled trial
SD: Standard deviation
VA: Veteran’s Affairs
VR-36: Veteran’s RAND 36 Item Health Survey
6MWT: 6-Minute walk test
